# Incidence of Depression Among Community Dwelling Healthy Elderly and the Predisposing Socio-environmental Factors

**DOI:** 10.7759/cureus.4292

**Published:** 2019-03-21

**Authors:** Maham Fatima, Alina Sehar, Maha Ali, Asra Iqbal, Faizan Shaukat

**Affiliations:** 1 Internal Medicine, Jinnah Sindh Medical University, Karachi, PAK; 2 Internal Medicine, United Medical and Dental College, Karachi, PAK; 3 Surgery, Dow University of Health Sciences, Karachi, PAK; 4 Internal Medicine, Jinnah Postgraduate Medical Center, Karachi, PAK

**Keywords:** depression, elderly, geriatric, pakistan

## Abstract

Introduction

The average life expectancy is increasing with better medical facilities and evolving technology. The ratio of the geriatric population is increasing, which drives a need to invest more in the physical and psychological health of older people. The aim of this study was to assess the incidence of depression in community-dwelling healthy older adults.

Methods

Geriatric Depression Scale-15 (GDS-15) was utilized in this study. Community-dwelling older adults (age 65+ years) were approached in parks, mosques, grocery stores, and waiting areas of banks. Demographic information including age, gender, education, marital status, employment, and financial status, and family structure was recorded. Data were entered and analyzed using SPSS v. 22.0.

Results

Out of the 367 elderly participants, there were 165 (45%) men and 202 (55%) women. Depression was reported in 37% (*n* = 136) individuals; 29.7% (*n *= 109) were suggestive of depression, while 33.2% (*n *= 122) were not depressed. Risk factors for the development of depression included female gender, not living with a spouse (separated/spouse died/single), being financially dependent, being employed, and living alone (not in a joint or nuclear family).

Conclusion

The incidence of depression is high among healthy community-dwelling elderly individuals in Pakistan. Geriatric health should be taken into consideration. There should be strategies and guidelines to screen the geriatric population for psycho-social symptoms and provide them with psychological counseling.

## Introduction

One of the major changes that the recent decades have seen is an increase in average life expectancy due to evolving knowledge and practices especially in the field of medicine. These changes have led to the rise in the ratio of the elderly population, causing a shift in the average age of population from younger to older [1-2]. According to a report published by the United Nations, in 2009, 6.1% of the Pakistani population was elderly (60+ years), which is estimated to grow to 14.9% by 2050 [3]. An increase in the ratio of the aged population has led to an increased burden of not only chronic diseases and physical disabilities but also of psychological illnesses [4]. Depression, a major global public health problem, is also common in the elderly. Depression in the elderly is responsible for a huge burden on the health sector. World Health Organization (WHO), in 2017, proposed 7% of the global elderly population to be depressed; however, depression is both underdiagnosed and undertreated in primary care settings [5]. Statistics regarding the physical and mental health of the geriatric population is limited. Even in Pakistan, not many studies have reported the incidence of depression in the elderly. Two previous studies conducted in Karachi, in 2007 and 2018, reported the prevalence of depression as 20% to 23% [6-7].

Depression should not be considered as a normal process associated with aging and it should be termed as a serious public health problem. The risk factors responsible for depression in the elderly can range from socio-environmental to interpersonal and psychological factors. Depression worsens the outcome and prognosis of chronic diseases in this age group, results in physical and psychological impairment, increases healthcare expenditure, and affects overall life expectancy [8].

The objective of our study was to determine the incidence of depression and its association with socio-environmental factors in the elderly living in Karachi, Pakistan.

## Materials and methods

It was a cross-sectional, observational study which utilized non-probability, convenient sampling technique. The study duration was from July to December 2018. It included community-residing elderly individuals in Karachi. Individuals of age 65 and above of both genders were included in the study [9]. With an estimated 40% prevalence of depression in this population, 95% level of the confidence interval and absolute precision of 0.05, the sample size calculated was 369 [10]. Four hundred participants were approached to participate in the study. Eight elderly persons refused to participate and responses of 25 individuals were excluded due to incomplete information. Hence, 367 respondents completed the study. Elderly people in recreational places such as parks, places of worship including mosques, grocery stores, and waiting areas of local banks were included in the study after informed consent. The persons visiting these places by themselves were more likely to be responsive, alert, and have conscious thought-process. Only individuals who could read and understand English were included. In order to exclude bias, elderly with known co-morbidities including diabetes, hypertension, dementia, or any other chronic illness, and institutionalized elderly (in hospitals and old age homes) were not included.

A questionnaire with two parts was created. The first part included socio-demographic characteristics of the participants including age, gender, education, marital status, employment, and financial status, and family structure. In Pakistan, two types of family structures are common: a nuclear family that comprises the parents and their dependent children or only parents with no dependent/independent children and a joint family where two or more nuclear families live together [11].

The second part of the questionnaire consisted of a standard instrument - Geriatric Depression Scale-15 (GDS-15) - to evaluate depression. It comprises 15-items, each has to be answered with either a “Yes” or a “No.” For every “Yes” one point was added to the score. A score of >5 was regarded as “suggestive of depression,” score of ≥ 10 was regarded as “almost always indicative of depression”. GDS-15 is a comprehensive scale, takes 5-7 minutes and is easy to administer in ill as well as healthy elderly persons [12]. The clinical utility of GDS-15 for depression screening and its sensitivity and specificity has been established in the literature. GDS-15 sensitivity was 81.3% (95% CI = 77.2% to 85.2%) and specificity = 78.4% (95% CI = 71.2% to 84.8%). [13].

SPSS for Windows, version 22.0 (Armonk, NY: IBM Corp.) was utilized for data entry and analysis. Frequencies and percentages were calculated for demographic data such as gender, marital status, employment status, family status, and the dependence status. GDS-15 scores were computed and categorized into “normal,” “suggestive of depression,” and “indicative of depression.” GDS-15 scores were compared with the socio-demographic characteristics of the participants.

## Results

Out of the 367 elderly participants, there were 165 (45%) men and 202 (55%) women. The socio-demographic characteristics of the participants are given in Table 1, which shows that the most common age group was 71-80 years (*n *= 173; 47.1%). More than half of the study sample was married and living with their spouses (*n* = 200; 54.4%). As far as the education status of the study sample was concerned, 129 (35.1%) were educated until the graduate/diploma level. The employment and financial status and the family structure of the participants were comparable, as seen in Table 1.

**Table 1:** Socio-demographic characteristics of the study participants

Socio-demographic Characteristics	Frequency n (%) (N=367)
Gender	
Male	165 (45%)
Female	202 (55%)
Age	
65–70 years	101 (27.5%)
71–80 years	173 (47.1%)
80+ years	93 (25.3%)
Mean ± SD	66.25 ± 9.58
Marital status	
Single/spouse died	139 (37.8%)
Married (living with spouse)	200 (54.4%)
Divorced	28 (7.6%)
Education status	
Illiterate	27 (7.3%)
Primary	43 (11.7%)
Secondary	31 (8.4%)
Intermediate	57 (15.5%)
Graduate / diploma	129 (35.1%)
Postgraduate	80 (21.8%)
Employment status	
Employed /self-employed	189 (51.5%)
Stay at home/ unemployed	178 (48.5%)
Financial status	
Independent (self-earning / pension / savings)	213 (58.1%)
Dependent (on children or others)	154 (41.9%)
Family structure	
Joint family (children and/or grandchildren)	134 (36.5%)
Nuclear family (with spouse only)	182 (49.5%)
Living alone	51 (13.9%)

Mean GDS score was 10.23 ± 2.35. Classified according to the scoring of GDS-15, there were 136 (37%) with score ≥10 indicating depression. There were 122 participants (33.2%) who were not depressed (GDS score 0-5) and 109 individuals (29.7%) were suggestive of depression (GDS score >5). Individuals were divided into two groups: indicative of depression (*n *= 136; 37%) and suggestive of depression/normal (*n *= 231; 63%). Socio-demographic characteristics of the participants were compared for both these groups, as shown in Table 2. When gender was taken into consideration, depression was more common in females than males (46% vs. 26%); depression was most common in the age group 71-80 years (41.6%) and least common in individuals of 80+ years (28%). When three marital statuses were compared, it was seen that individuals who were single or whose spouse has died were most depressed (70.5%). When educational status was compared, it was seen that participants of secondary and intermediate level of education were more depressed than those educated till postgraduate level. The comparison of all socio-demographic characteristics is shown in Table 2​​​​​​​.

**Table 2:** Association of demographic characteristics with the incidence of depression among the community dwelling elderly Indicative of depression, GDS-15 score ≥10; suggestive of depression/normal, GDS-15 score <9 GDS, geriatric depression scale

Socio-demographic Characteristics	Indicative of depression	Suggestive of depression / normal
Gender		
Male	43 (26%)	122 (74%)
Female	93 (46%)	109 (54%)
Age		
65–70 years	38 (37.6%)	63 (62.4%)
71–80 years	72 (41.6%)	101 (58.4%)
80+ years	26 (28%)	67 (72%)
Marital status		
Single/spouse died	98 (70.5%)	41 (29.5%)
Married (living with spouse)	88 (44%)	112 (56%)
Divorced	16 (57.1%)	12 (42.9%)
Educational status		
Illiterate	10 (37%)	17 (63%)
Primary	11 (25.5%)	32 (74.5%)
Secondary	17 (54.8%)	14 (45.2%)
Intermediate	28 (49.1%)	29 (50.8%)
Graduate / diploma	56 (43.4%)	73 (56.6%)
Postgraduate	14 (17.5%)	66 (82.5%)
Employment status		
Employed / self-employed	88 (46.5%)	101 (53.5%)
Stay at home / unemployed	48 (27%)	130 (73%)
Financial status		
Independent (self-earning / pension / savings)	43 (20.2%)	170 (79.8%)
Dependent (on children or others)	93 (60.4%)	61 (39.6%)
Family structure		
Joint family (children and/or grandchildren)	34 (25.3%)	100 (74.7%)
Nuclear family (with spouse only)	97 (53.3%)	85 (46.7%)
Living alone	42 (82.3%)	9 (17.7%)

## Discussion

This study has reported striking results. The incidence of depression among healthy, community-dwelling elderly is relatively high. Females had a higher incidence of depression than males. Other factors associated with a higher incidence of depression were being single/divorced, the death of the spouse, financial dependence, being employed, and living alone.

The overall prevalence of depression in elderly reported in this study is comparable to other studies from this region [6-7,10]. Results from other countries such as Canada and Greece are also comparable [14-15].

Female gender has been associated with overall higher incidence of depression in the general population as well as in the geriatric group [14]. This could be because of female depression is associated with hormonal changes [14]. Pakistan is a collectivistic society with high cultural and family values. Joint and extended family systems are commonly seen here. The elderly in Pakistan live alone infrequently; most of the times they are looked after by younger members of the families as a part of their socio-cultural, religious, and moral obligation. Adults of the families provide economic support to their old parents [16-18]. The participants living in joint families and even in nuclear families were less depressed as compared to those living alone. This comes with cultural support, social interaction, and caregiving. However, the family system in urban Pakistan is evolving. More families and more young adults prefer living independently which will increase the percentage of older people living alone or ending up in nursing homes in the near future.

Peculiarly enough, although financially dependent elderly were more depressed in this study which is an established factor in other studies too [19], this study reported a higher incidence of depression among the employed elderly. Even in another multi-national analysis, elderly who retired with a pension had a protective factor depression in comparison to the elderly who were then employed [20]. Employment in older age indicates the need for money and lack of any financial support from family or friends. Furthermore, job stress is too much to handle for people of this age group [21].

Elderly individuals, who were single or separated from their spouses or whose spouses have died, showed greater depression as compared to those who were married and living with their spouses. This result was comparable to various other studies [22-23]. The grief of losing the spouse is debilitating for psychosocial health at all stages of life; the elderly are particularly vulnerable to this grief as their dependency and emotional attachment with the spouses increase with age [6].

The study has its limitations too. Firstly, it was a cross-sectional study design through which no formal relationship can be established. A more real picture of this population can be sketched by utilizing the Urdu translated version of GDS-15 as it will be more inclusive in this population.

## Conclusions

There is high prevalence of depression among elderly population in Pakistan. Geriatric health should be made a priority. There should be strategies and guidelines to screen geriatric population for psychosocial symptoms and provide them with psychological counseling. The changing parameters of family system in Pakistan should be taken into consideration by public health specialists and more time, research, and efforts should be invested in elderly well-being. Elderly individuals should be empowered enough to lead independent and satisfied lives. 
